# Total plasma *N*-glycomic signature of SARS-CoV-2 infection

**DOI:** 10.1016/j.isci.2024.110374

**Published:** 2024-06-24

**Authors:** Marco R. Bladergroen, Tamas Pongracz, Wenjun Wang, Simone Nicolardi, Sesmu M. Arbous, Anna Roukens, Manfred Wuhrer, BEAT-COVID group, LUMC COVID-19 group

**Affiliations:** 1 Center for Proteomics and Metabolomics, Leiden University Medical Center, Leiden 2333 ZA, the Netherlands; 2 Department of Intensive Care, Leiden University Medical Center, Leiden 2333 ZA, the Netherlands; 3 Department of Infectious Diseases, Leiden University Medical Center, Leiden 2333 ZA, the Netherlands

**Keywords:** Disease, Human, Components of the immune system, Glycomics

## Abstract

Total plasma protein *N*-glycosylation (TPNG) changes are a hallmark of many diseases. Here, we analyzed the TPNG of 169 COVID-19 patients and 12 healthy controls, using mass spectrometry, resulting in the relative quantification of 85 *N*-glycans. We found a COVID-19 *N*-glycomic signature, with 59 glycans differing between patients and controls, many of them additionally differentiating between severe and mild COVID-19. Tri- and tetra-antennary *N*-glycans were increased in patients, showing additionally elevated levels of antennary α2,6-sialylation. Conversely, bisection of di-antennary, core-fucosylated, nonsialylated glycans was low in COVID-19, particularly in severe cases, potentially driven by the previously observed low levels of bisection on antibodies of severely diseased COVID-19 patients. These glycomic changes point toward systemic changes in the blood glycoproteome, particularly involvement of acute-phase proteins, immunoglobulins and the complement cascade. Further research is needed to dissect glycosylation changes in a protein- and site-specific way to obtain specific functional leads.

## Highlights

• COVID-19 patients can be distinguished from controls by plasma *N*-glycans
• Severity of COVID-19 coincides with distinct glycosylation signatures
• α2,6-Sialylation is elevated in severe COVID-19

## Graphical abstract

**Figure:**
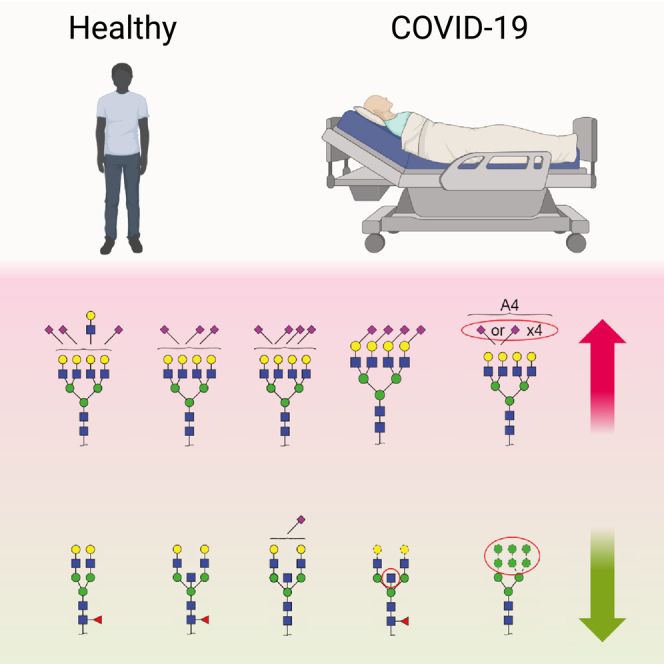

## Introduction

The first global pandemic of the 21^st^ century has been caused by the spread of severe acute respiratory virus 2 (SARS-CoV-2), a virus causing coronavirus disease 2019 (COVID-19). SARS-CoV-2 infections are characterized by divergent disease trajectories, the outcomes of which are difficult to predict early on.^1^^,^^2^^,^^3^ Various demographic factors and comorbidities have been associated with a higher risk of developing severe COVID-19 and are therefore considered key factors in clinical severity score calculations, but patients with severe COVID-19 are distinguished by an often unpredictable, rapidly intensifying inflammatory response.^1^^,^^3^^,^^4^ The case fatality rate of hospitalized patients spanned from 0.1 to 18.2%, while this number approached 42% for intensive care unit (ICU) admitted patients at the early stages of the pandemic.^5^^,^^6^ Hence, further predictive markers are of interest for more efficient patient stratification.

Various reports have shown that major immune dysregulation accompanies mild to moderate disease transitions and in particular severe cases.^4^^,^^7^^,^^8^^,^^9^ While these studies provided invaluable insights into COVID-19 disease mechanism, they largely, but not exclusively, rely on immune cell-associated biomarkers. Indeed, being a respiratory tract infection, COVID-19 leads to alveolar damage due to both viral infiltration and concomitant dysfunctional host immune responses.^4^ However, extrapulmonary complications such as coagulation disorders and even liver injuries have been reported as prevalent in patients with severe COVID-19 in comparison to those with a mild disease.^10^^,^^11^^,^^12^

**Figure 1:**
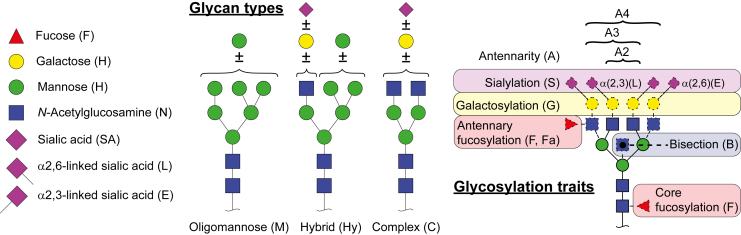
Schematic representation of monosaccharides, glycan types and glycosylation traits Dashed monosaccharides are optional features.

Severe COVID-19 has been found to come with major systemic changes affecting not only the plasma metabolome^13^ but also the proteome^14^^,^^15^ showing distinct signatures. Importantly, most blood proteins are synthetized and glycosylated in the liver. Glycosylation is a common post-translation modification with versatile and profound effects on protein structure and function.^16^^,^^17^ These glycans are characterized by structural motifs such as fucosylation, sialylation and antennarity (glycosylation traits) that reflect their biosynthesis and function. A schematic representation of glycans and glycosylation traits is depicted in Figure 1.

In the context of COVID-19, glycomics studies have largely focused on circulatory immunoglobulin G (IgG) glycosylation, which has been associated with disease severity,^18^^,^^19^^,^^20^^,^^21^ but these changes reflect only alterations in the glycosylation machinery of B cells. On the other hand, changes of total plasma protein *N*-glycosylation (TPNG, i.e., enzymatically released *N*-glycans from all plasma proteins) have been described in cancers, autoimmune diseases as well as infectious diseases, but also in inflammatory diseases affecting the liver.^22^^,^^23^^,^^24^^,^^25^^,^^26^^,^^27^^,^^28^ With regard to TPNG changes in COVID-19, an *N*-glycan with the composition 5 hexoses (H), 5 N-acetylhexosamines (N), and 1 fucose (F) – presumably corresponding to an immunoglobulin G-related di-antennary, di-galactosylated, core-fucosylated and bisected structure – negatively correlated with disease severity.^29^ Two other studies indicated increased antennarity and tetra-sialylation in the TPNG of COVID-19 patients.^30^^,^^31^ Regarding the relative abundance of oligomannose glycans in TPNG, findings were disparate, with respect to both the glycans observed, and the COVID-19-associated effects.^15^^,^^29^^,^^30^^,^^31^ In another study, increased α2,6-sialylation of complement factors has been described in patients’ plasma and lung tissue.^32^

Here, we report on the analysis of total plasma *N*-glycosylation in a longitudinal, observational cohort of hospitalized SARS-CoV-2 infected individuals. Total plasma *N*-glycosylation in COVID-19 was studied at unprecedented depth by employing an established, semi-automated and high-throughput workflow involving the enzymatic release, linkage-specific sialic acid derivatization and subsequent hydrophilic interaction-based purification of *N*-glycans, followed by their analysis employing matrix-assisted laser desorption/ionization Fourier transform ion cyclotron resonance mass spectrometry (MALDI-FTICR-MS).^33^^,^^34^ Our aim was to explore the total plasma *N*-glycome as a potential source of non-invasive markers for severe COVID-19 development in hospitalized patients with varying disease severity. Therefore, we compared the obtained signatures across the clinical severity spectrum, and also compared patients’ signatures to those of healthy controls.

## Results

MALDI-MS analysis of the total plasma *N*-glycome from longitudinal plasma samples of 169 patients and 12 healthy controls (participant characteristics summarized in Table 1) allowed the identification of overall 85 *N*-glycans from which 48 glycosylation traits were calculated (Tables S1, S2, and S3).

**Table 1:** Cohort characteristics

	Controls (*n* = 12)	Patients (*n* = 169)	ICU (T0, *n* = 68)	non-ICU (T0, *n* = 85)
Age - median (IQR)	64 (62–65)	66 (58–72)	66 (60–71)	66 (54–75)
Female (%)	4 (33.3)	43 (25.4)	15 (22.1)	22 (25.9)
Male (%)	8 (66.6)	126 (74.6)	53 (77.9)	63 (74.1)
Severity score - median (IQR)	–	6 (3–12)	12 (10–14)	3 (2–5)
Days since onset - median (IQR)	–	13 (10–17)	15 (12–19)	11 (9–14)

Principal Component Analysis using all glycans and glycosylation traits indicated a severity-driven separation among the patients (Figure S1). Patients with low disease severity were clustering with the healthy controls as well as with the plasma pool while severe patients were noticeably separated. Inspection of the loadings plot suggested that this separation reflecting disease severity was largely driven by tri- and tetra-antennary glycans.

### Glycomic profiles distinguish COVID-19 patients from controls

**Figure 2:**
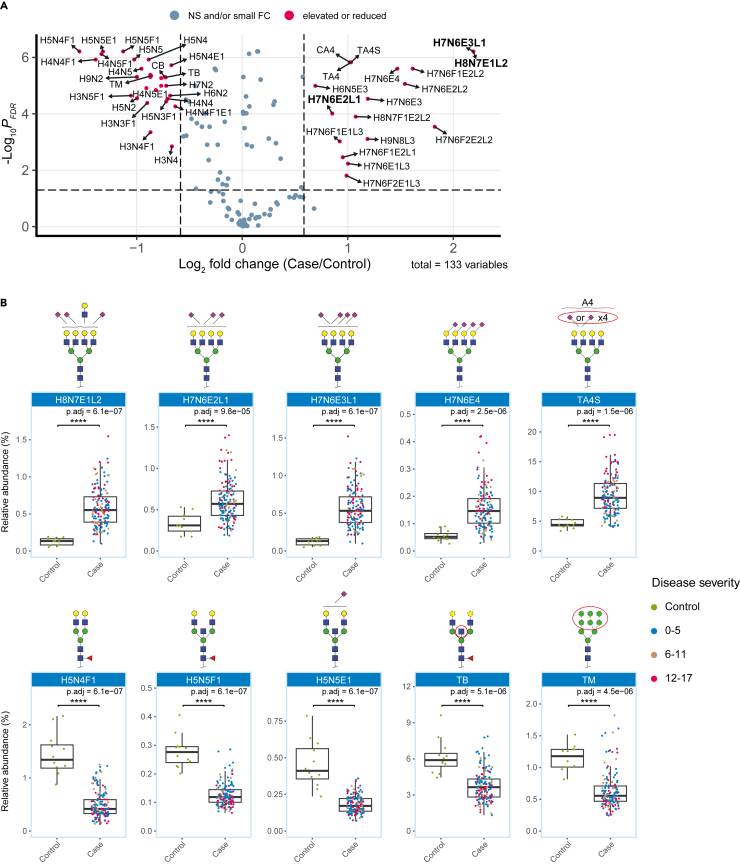
Differentially expressed glycans and glycosylation traits in COVID-19 patients at the time of hospital admission (T0) vs. controls (A) Volcano plot showing the significantly elevated and reduced glycans and glycosylation traits in COVID-19 patients compared to healthy individuals at a fold change cut-off of 1.5 (dashed lines on x axis) and p_FDR_-value of <0.05. Glycans in bold were selected by the ROC analysis (Figure S2). (B) Altered glycans and glycosylation traits as deducted from panel A and Figure S1, colored according to categorized disease severity. The error bars indicate variability outside the first and third quantiles (white boxes) around the median (bold midline). *P*-values are based on Wilcoxon rank-sum tests after Benjamini-Hochberg multiple testing correction (p.adj). ∗: p.adj<0.05, ∗∗: p.adj<0.01, ∗∗∗: p.adj<0.001, ∗∗∗∗: p.adj<0.0001. Concurrent glycan structures are putative and may comprise additional isomers. Dashed lines in these structures represent optional building blocks. See also Figures S1 and S2.

To further investigate which glycosylation features convey distinctive power, glycosylation profiles of patients at hospitalization (T0) were compared to those of healthy controls. Most of the analyzed glycans and glycosylation traits appeared to be significantly altered in COVID-19 patients as compared to controls. Overall, 57 of the 85 glycans, and 34 of the 48 glycosylation traits were found to be different (Table S4). To narrow down our focus to diagnostically meaningful signatures, only glycans and glycosylation traits with at least 1.5-fold difference between the two groups were considered in subsequent analyses (Figure 2).

From the selected features, tri- and tetra-antennary glycans were found to be elevated, while di-antennary and bisected glycans were decreased in patients relative to healthy controls. Specifically, tetra-antennary glycans (TA4) showed a median relative abundance of 9.4% in patients vs. 4.6% in controls (Table S4). Intriguingly, COVID-19 patients showed elevated levels of sialylation on a range of glycans: Sialylation of di-antennary *N*-glycans (A2S) was elevated in patients (87%) relative to controls (79%) as was sialylation of tri-antennary (A3S) and tetra-antennary *N*-glycans (A4S). The linkage-specific derivatization allowed us to dissect the contribution of α2,6-and α2,3-sialylation to these high sialylation levels: α2,3-sialylation of tetra-antennary *N*-glycans (A4L) was 48% for both COVID-19 patients and controls, whilst α2,6-sialylation of tetra-antennaries (A4E) was specifically elevated in COVID-19 patients (41% vs. 36%; *p* = 6.1e-6; Table S4). Accordingly, particularly pronounced differences were seen for tri- and tetra-antennary glycans with three or four α2,6-linked sialic acids (H6N5E3, H7N6E3, and H7N6E4; Figure 2; Figure S2A), together with various other α2,6-sialylated tri- and tetra-antennary glycans, with all of these being elevated in patients. Conversely, di-antennary bisected and fucosylated glycans including H5N5F1 (0.3% vs. 0.1%), H4N5F1 (0.8% vs. 0.3%), and H3N5F1 (0.4% vs. 0.2%) were decreased in COVID-19. Likewise, the relative abundance of oligomannose glycans (TM; 1.2% vs. 0.6%) was found to be lower in most cases (Figure 2B).

### Predictive model highlights *N*-glycans with highest classification accuracy in differentiating patients from controls

Next, we explored the potential of plasma *N*-glycans for discriminating COVID-19 patients at baseline from healthy controls. Receiver Operating Characteristics (ROC) analysis showed two related models, both including H7N6E2L1 supplemented with either H8N7E1L2 or H7N6E3L1 (E − 2,6-linked sialic acid; L – 2,3-linked sialic acid), differentiating patients from controls (Figure S2B; Table S5). When performing a similar analysis considering samples at highest disease severity (TH, Figure S3), we found H5N4F1, H8N7E1L2 and H7N6E3L1 as the differentiating glycans, in overlap with the baseline findings. Interestingly, the models at both T0 and TH included H8N7E1L2 or H7N6E3L1, and ROC analysis on the individual glycans indicated that each of these two alone was sufficient to distinguish between patients and controls with similar performance as the models as deduced by the SES algorithm (Table S5). Some caution is advised though, since the number of controls is limited with possibly a slight overfitting as result. Nonetheless, the results are of sufficient strength for a discriminating test. Furthermore, the T0 state is already very varied between patients, both in moment of hospitalization as well as in severity and pre-disease state is unknown. A large sample overlap between T0 and TH is apparent.

### Glycosylation signatures reflect disease severity

**Figure 3:**
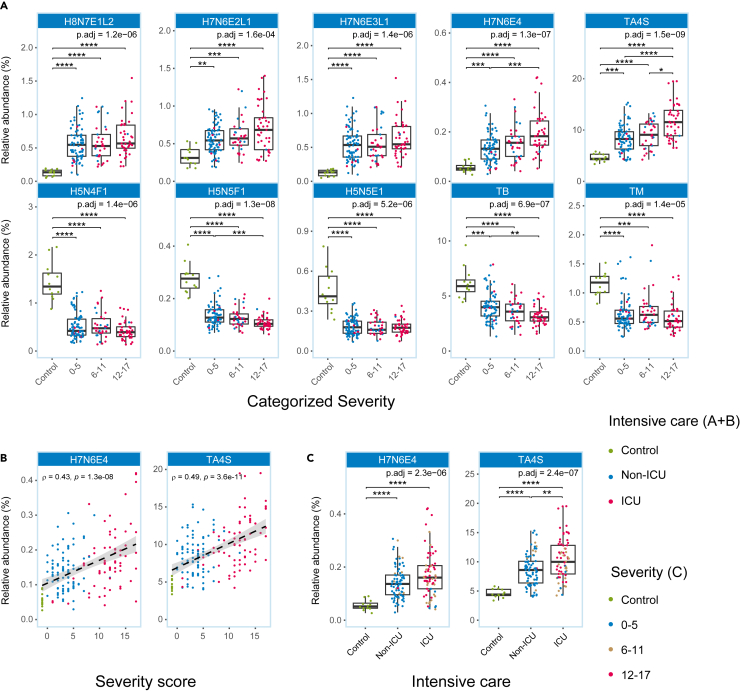
Glycan expression coinciding with severity (A) Boxplots showing differential glycan expression with severity at time of hospital admission (T0). The error bars indicate variability outside the first and third quantiles (white boxes) around the median (bold midline). *p*-values are based on Kruskal-Wallis tests with post-hoc Dunn’s test after Benjamini-Hochberg multiple testing correction (p.adj). ∗: p.adj<0.05, ∗∗: p.adj<0.01, ∗∗∗: p.adj<0.001, ∗∗∗∗: p.adj<0.0001. (B) Correlation plots of the two best performing analytes. Black dashed lines are based on loess regression, gray area represents the 95% confidence interval. Rho- and *p*-values are based on Spearman correlation. (C) Boxplots of the two analytes in (B) according to ICU admission. The individual samples in the plots are represented by circles, colored according to either intensive care admission (A and B) or categorized severity (C). Intensive care admission is defined as being admitted to the intensive care unit (ICU) at any point in time during COVID-19 infection, not specifically for T0.

We next explored the association of glycosylation with disease severity focusing on the glycans and glycosylation traits which were found to be major discriminators in the cross-sectional analysis comparing healthy with diseased. We observed that glycans representing high levels of sialylation of tri- and tetra-antennary glycans were elevated across all three disease severity groups of COVID-19 patients compared to controls (H8N7E1L2; H7N6E2L1; H7N6E3L1 and H7N6E4) and the same held true for the overall sialylation of tetra-antennary glycans (TA4S; Figure 3A). In line with the cross-sectional analysis (Figure 2), di-antennary, bisected glycans (H5N4F1, H5N5F1, H5N5E1; TB) as well as levels of oligomannose glycans (TM) were lowered in all three disease severity groups (Figure 3A). We next explored the correlation of glycosylation traits with disease severity and observed that TA4S and H7N6E4 showed the strongest correlation with disease severity (Figure 3B). Accordingly, H7N6E4 as well as TA4S were higher in patients with ICU admission as compared to those who were not admitted to ICU (Figure 3C). Of note, H7N6E4 and TA4S showed high levels in high-severity cases over the entire period of hospitalization, consistently reflecting high disease severity (Figure S4).

As many of the glycoproteins in plasma are liver-derived, including those carrying tri- and tetra-antennary complex-type glycans,^16^ we aimed to assess whether the severity-associated glycosylation features would associate with alanine aminotransferase (ALAT) and aspartate aminotransferase (ASAT) levels, which are established markers of liver damage. The glycosylation trait TA4S associated with ASAT levels (ρ = 0.38; *p* = 5.9e-5; Table S6) indicating that severity-associated glycosylation changes may in part reflect liver damage.

### Association of bisection with disease severity is largely driven by IgG bisection

**Figure 4:**
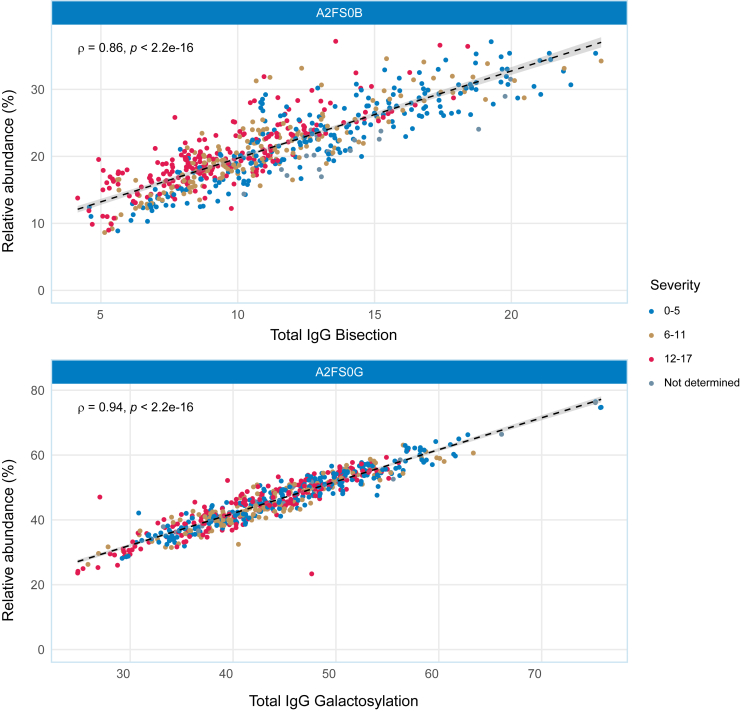
Total plasma bisection and galactosylation correlate with IgG bisection and galactosylation Individual samples colored according to categorized severity, indicating lower bisection and lower galactosylation with higher severity. Black dashed lines are based on loess regression, gray area represents the 95% confidence interval. Rho- and *p*-values are based on Spearman correlation.

Di-antennary, fucosylated, non-sialylated *N*-glycans in TPNG are considered to be largely IgG-derived.^16^ Accordingly, we found that the level of bisection as well as the level of galactosylation of di-antennary, fucosylated, asialylated *N*-glycans correlate very well with total IgG bisection and Total IgG galactosylation respectively (Figure 4), determined in our previous study on IgG glycosylation of COVID-19 patients.

## Discussion

Here we determined the plasma *N*-glycomic signature of COVID-19 by mass spectrometry relatively quantifying a set of 85 glycans and 48 glycosylation traits. Our analysis of the COVID-19-associated TPNG signatures is considerably deeper than previous studies,^15^^,^^29^^,^^30^^,^^31^^,^^35^ as we more than doubled the coverage of glycan structures and covered glycans at higher mass range with sialyl-linkage isomers resolved. The COVID-19 TPNG was remarkably different from the TPNG of healthy controls already at the moment of hospital admission, and the relative abundance of the single glycans H8N7E1L2 and H7N6E3L1 both allowed a near perfect separation of the patient and control groups (Figure S2).

Most of the tri- and tetra-antennary *N*-glycans were found to be higher in cases than in controls. These, for a large part highly sialylated glycans, likely stem from acute-phase proteins and may reflect an increase of acute-phase protein concentrations in the blood of COVID-19 patients.^15^ Specifically, highly sialylated tetra-antennary *N*-glycans in TPNG may for a large part come from alpha-1 acid glycoprotein^16^ which shows highly elevated plasma levels in hospitalized COVID-19 patients (167 mg/dL) as compared to healthy controls (69 mg/dL).^15^ Furthermore, such glycans have been associated with the complement cascade^32^ and an (over)activation of this pathway is believed to increase viral disease severity via a strong pro-inflammatory response (reviewed by Mellors et al.^36^). Reduction of the pro-inflammatory effects by inhibition of the C5a receptor resulted in a lower disease severity in hDPP4-transgenic mice infected with MERS-CoV.^37^ Complement-mediated pathology has also been observed in tissue of SARS-CoV-2 infected individuals.^38^^,^^39^

While the overall levels of sialylation were increased across di-, tri- and tetra-antennary *N*-glycans, this appeared to be exclusively driven by an increase in α2,6-sialylation. Reference data on TPNG profiles of other acute infections or pulmonary conditions are lacking, although increased levels of highly branched, highly sialylated structures have been observed in chronic obstructive pulmonary disease (COPD) correlating with disease severity,^40^ the linkage-type of the sialylation was not determined. When looking more generally at prevalent disease, elevated α2,6-sialylation has been reported for both type-2 diabetes (T2D)^24^ and colorectal cancer (CRC).^23^ Interestingly, T2D and CRC also feature low levels of oligomannose glycans (TM), similar to our results for COVID-19. The lowered TM in COVID-19 may be a consequence of the enhanced clearance of TM-containing glycoconjugates via the mannose receptor (CD206) which has been found to be highly expressed on alveolar macrophages in lung inflammatory conditions associated with the disease.^41^

Furthermore, we found that bisection of di-antennary, fucosylated, asialylated *N*-glycans (H3N5F1, H4N5F1 and H5N5F1) were down in COVID-19 to only approx. half the levels found for controls. In line with Paton et al., we found that H5N5F1 levels were lower in those with very severe COVID-19 compared to the ones with lower disease severity (Figure 3).^29^ This observation of a decrease of H5N5F1 in TPNG with increasing disease severity is in line with our previous analysis of total IgG1 Fc glycosylation of the same patients, with IgG1 Fc bisection negatively associating with disease severity.^18^ There, we found total IgG1 Fc bisection to be down to 10% in the highest disease severity group, and found anti-S IgG1 to show an even more skewed phenotype, with a mere 5% bisection in the highest disease severity group. One may speculate, therefore, that the decreased levels of bisection of di-antennary, fucosylated, non-sialylated *N*-glycans in TPNG may in part be driven by a low-bisection COVID-19-specific antibody response.

Similar to COVID-19, the two inflammatory bowel disease (IBD) variants ulcerative colitis (UC) and Crohn’s disease (CD) likewise display low bisection of core-fucosylated, non-sialylated di-antennaries – in contrast to other prevalent diseases such as CRC but also T2D and COPD which show elevated levels of this feature.^23^^,^^40^ Interestingly, mono-galactosylated di-antennary structures, including bisected ones, were identified as most prominently decreased in both plasma and IgG *N*-glycome of COPD patients.^40^ We observed a significant reduction in the relative abundances of these glycans in COVID-19 patients as well.

Changes of total plasma protein *N*-glycosylation have been described in inflammatory diseases affecting the liver, and liver injuries have been reported as prevalent in patients with severe COVID-19 in comparison to those with a mild disease. Our data indicate a minor relationship between COVID-19 and liver injuries (Table S6). Most significant correlation between glycans and liver injury markers ALAT and ASAT occurred with sialylated tri-and tetra-antennary glycans including the disease severity-associated glycosylation trait TA4S. These associations indicate that the COVID-19 severity-associated glycosylation traits may in part reflect liver damage.

Several glycosylation changes discussed here are typical signatures of an inflammatory response. In this respect we would like to mention the elevated levels of α2,6-linked sialic acids as well as reduced levels of galactosylation of fucosylated, non-sialylated, di-antennary glycans (A2FS0G; Figure 4B), the latter generally being linked to IgG glycosylation which showed reduced levels in a number of diverse diseases.^42^ Therefore, we cannot exclude the possibility that the observed glycosylation profiles are not COVID-19 specific but represent more general phenotypes.

While it is well-established that different diseases exhibit different TPNG signatures,^23^ further research is needed to dissect the causes and mechanisms leading to these glycosylation patterns, ideally resolving protein- and site-specific contributions to these signatures. Furthermore, the functional relevance of plasma protein glycosylation changes deserves attention. Whilst for IgG Fc glycosylation quite some functional implications are known,^43^ such knowledge is largely lacking for the rest of the plasma proteins.

### Limitations of the study

Our study has several limitations. The number of controls is low, certainly compared to the number of enrolled COVID-19 patients and the time span for the control individuals was much shorter than that of the patients. For the COVID-19 patients no pre-disease samples were available, and we therefore cannot conclude whether the observed glycosylation signatures were COVID-19 induced or rather already present prior to the disease. Isomers were in part resolved using linkage-specific sialic acid derivatization,^34^ yet other isomers were not resolved. Glycosylation was studied at the released glycan level, and no information on protein carriers and glycosylation sites was obtained. Future studies should evaluate the functional significance of COVID-19, and in general terms disease-associated glycosylation changes.

## Consortia

**BEAT-COVID group (in alphabetical order):** S.M.A., Bernard M. van den Berg, Suzanne Cannegieter, Christa M. Cobbaert, Anne M. van der Does, Jacques J.M. van Dongen, Jeroen Eikenboom, Mariet C.W. Feltkamp, Annemieke Geluk, Jelle J. Goeman, Martin Giera, Thomas Hankemeier, Mirjam H.M. Heemskerk, Pieter S. Hiemstra, Cornelis H. Hokke, Jacqueline J. Janse, Simon P. Jochems, Simone A. Joosten, Marjolein Kikkert, Lieke Lamont, Judith Manniën, Tom H.M. Ottenhoff, T.P., Michael R. del Prado, Núria Queralt Rosinach, Meta Roestenberg, Marco Roos, A.R., Hermelijn H. Smits, Eric J. Snijder, Frank J.T. Staal, Leendert A. Trouw, Roula Tsonaka, Aswin Verhoeven, Leo G. Visser, Jutte J.C. de Vries, David J. van Westerloo, Jeanette Wigbers, Henk J. van der Wijk, Robin C. van Wissen, M.W., Maria Yazdanbakhsh, Mihaela Zlei.

**COVID-19 group (in alphabetical order):** S.M.A., Meryem Baysan, Mark G.J. de Boer, Anske G. van der Bom, Olaf M. Dekkers, Anna M. Eikenboom, Susan B. ter Haar, Laura Heerdink, Lieke J. van Heurn, Ingeborg de Jonge, Willem Lijferink, Romy T. Meier, Josine A. Oud, Frits Rosendaal, Alexandra G.L. Toppenberg, Jonathan W. Uzorka, Annekee A. van IJlzinga Veenstra, Jeanette Wigbers, Julia M. Wubbolts.

## STAR★Methods

### Key resources table

**Table:** 

REAGENT or RESOURCE	SOURCE	IDENTIFIER
**Biological samples**		

Sera from individuals presenting with COVID-19.	Patients hospitalized at the Leiden University Medical Center in 2020	N/A
Sera from healthy volunteers.	Recruited at the Leiden University Medical Center in 2020	N/A

**Chemicals, peptides, and recombinant proteins**		

PNGaseF	Merck, Darmstadt, Germany	Cat#11365177001

**Deposited data**		

RAW, analyzed and meta data	https://glycopost.glycosmos.org/	GlycoPOST: GPST000400

**Software and algorithms**		

Rstudio	Rstudio Team^44^	https://posit.co/
MXM R-package	Lagani et al.^45^	https://cran.r-project.org/package=MXM
MassyTools	Jansen et al.^46^	https://git.lumc.nl/cpm/massytools

**Other**		

Hamilton STAR and STARplus robotic system	Hamilton	https://www.hamiltoncompany.com/automated-liquid-handling
Bruker solariX 15T FT-ICR-MS	Bruker	https://www.bruker.com/en/products-and-solutions/mass-spectrometry/mrms/solarix.html

### Resource availability

#### Lead contact

Further information and requests for resources and reagents should be directed to and will be fulfilled by the lead contact, Marco R. Bladergroen (m.r.bladergroen@lumc.nl).

#### Materials availability

This study did not generate new unique reagents.

#### Data and code availability

• Raw mass spectrometry data in the form of .xy files have been deposited at GlycoPost^47^ and are publicly available as of the date of publication. Accession numbers are listed in the key resources table. This repository also contains the relevant metadata as well as the final curated data (Table S1) used in this manuscript.
• This paper does not report original code.
• Any additional information required to reanalyze the data reported in this work paper is available from the lead contact upon request.

### Experimental model and study participant details

This work was limited to human subjects. The total number of 181 participants (patients and controls) was divided into a 47:134 ratio female:male. Throughout the text, when sex is mentioned, the term sex assigned at birth is meant. Age ranged from 18 to 91 years. All participants originated from the Netherlands. Other racial or ethnic information was not registered. More details are presented in Table 1. All available metadata per patient is also included in Table S1. This study has been approved by the Medical Ethics Committee Leiden-Den Haag-Delft (NL73740.058.20), complied with the latest version of the Declaration of Helsinki, and an informed consent was obtained in all cases. The trial was registered in the Dutch Trial Registry (NL8589).

### Method details

#### Patient recruitment and sample collection

Plasma samples from 169 hospitalized COVID-19 positive patients, named “cases” for the purpose of this study, were collected over a period of 9 months (between May 2020 and December 2020) during the first and second wave of the COVID-19 pandemic, as a prospective, observational study within the Leiden University Medical Center, known as the BEAT-COVID cohort. Additionally, samples, already available from the local biobank, from 3 patients later proven to be COVID-19 positive were added to this cohort, together with 12 healthy individuals (5 samples each during a one-month sampling regime) (Tables 1 and S1). None of the participants had received a COVID-19 vaccine. Serial samples were collected during the entire hospitalization period of the patients including a single sample six weeks after release from the hospital for 24 patients. Sample size was based on availability. For some patients, a sample at hospital admission/baseline (T0) was not available, either by absence in our sample set or by rejection in data curation due to poor spectral quality. Therefore, the sum of ICU and non-ICU patients at baseline does not match the total number of patients included. A patient is categorized as ICU when admitted to the ICU anytime during hospitalization, not necessarily at hospital admission/baseline (T0).

#### Chemicals and reagents

Analytical grade ethanol, sodium dodecyl sulfate (SDS) and trifluoroacetic acid (TFA) were purchased from Merck (Darmstadt, Germany). Disodium hydrogen phosphate dihydrate, potassium dihydrogen phosphate sodium chloride, 85% phosphoric acid, 50% sodium hydroxide, nonidet P-40 substitute (NP-40), 1-hydroxybenzotriazole 97% (HOBt) and super-DHB (9:1 mixture of 2,5-dihydroxybenzoic acid and 2-hydroxy-5-methoxybenzoic acid, sDHB) were obtained from Sigma-Aldrich (Steinheim, Germany). 1-Ethyl-3-(3-(dimethylamino)propyl) carbodiimide (EDC) hydrochloride was purchased from Fluorochem (Hadfield, UK), whereas recombinant peptide-N-glycosidase F (PNGase F) was purchased from Roche Diagnostics (Mannheim, Germany). HPLC-grade acetonitrile (ACN) was obtained from Biosolve (Valkenswaard, The Netherlands) and Type-I ultrapure water was generated from a Q-Gard 2 system (Milli-Q or MQ, Millipore, Amsterdam, The Netherlands) in case of feed to the automated sample processing system (for rinsing of reusable tips) or by an ELGA Purelab Ultra system (Elga LabWater, High Wycombe, United Kingdom) to create solutions. The cotton thread used for HILIC purification was obtained from Pipoos (Utrecht, The Netherlands).

#### Sample preparation and mass spectrometry

The samples were distributed over 96-well plates, together with technical replicates of a commercially available plasma standard pool (VisuCon-F: Affinity Biologicals (Ancaster, ON, Canada) as well as blanks (PBS), to monitor the quality of the glycomic sample preparation and measurements. Glycans were released from proteins as described previously.^34^ In short, after denaturation of 6 μL of serum with 12 μL 2% SDS at 60°C for 10 min, *N*-glycans were enzymatically released from plasma glycoproteins using a fresh release mixture (6 μL of 4% NP-40, 6 μL of acidified PBS (100 mM phosphoric acid in 5X PBS) and 0.6 μL of PNGase F) for each sample and incubated overnight at 37°C.

Released glycans were subjected to chemical derivatization resulting in stabilized and distinguishable α2,3- and α2,6-sialic acid linkage isomers. Derivatized *N*-glycans were purified and spotted onto a MALDI-target plate using the previously described automated liquid handling platform.^33^ For the chemical derivatization, 2 μL of the released glycan mixture was added to 40 μL of esterification reagent, which consisted of freshly prepared 0.25 M EDC and 0.25 M HOBt dissolved in 100% ethanol, followed by incubation for 1 h at 37°C.^48^ Subsequently, 40 μL of acetonitrile was added. For the subsequent purification, in-house prepared cotton HILIC microtips were used (approx. 3 mm, 180 μg cotton thread per tip). The tips were pre-washed with ultrapure water and 85% acetonitrile to remove contaminants. To achieve glycan retention on the cotton HILIC stationary phase, samples in 85% ACN were carefully pipetted up and down 20 times. The tips were first washed with 85% acetonitrile containing 1% TFA followed by 85% acetonitrile, and finally eluted in 20 μL water. Subsequently, 7 μL of the purified sample plus 7 μL of sDHB matrix (2.5 mg/mL in 50% ACN with 0.1 mM NaOH)^49^ was premixed in a 384-well plate. Then, 2 μL of the mixture was spotted onto a MALDI target plate (800/384 MTP AnchorChip, Bruker Daltonics, Bremen, Germany), and after drying the spotted samples were directly analyzed by MALDI-FTICR-MS.

MALDI-FTICR-MS was performed on a Bruker 15T solariX XR FTICR mass spectrometer equipped with a CombiSource and a ParaCell (Bruker Daltonics). The system was controlled by ftmsControl version 2.1.0 and spectra in a *m/z*-range from 1011.2 to 5000.00 with about 1.7 million datapoints per spectrum were recorded. The instrument was calibrated once at the beginning of each full-plate measurement using CsI clusters.^50^ Each single spectrum was generated from 10 scans of 200 laser shots per raster point within the sample spot.

#### Data processing

DataAnalysis 5.0 SR1 (build 203.2.3586, Bruker Daltonics) was used to visualize and export MALDI-FTICR-MS spectra into text (.xy) file format. A list of 124 glycan compositions (including two dummy glycans) (Table S2) were used to extract peak areas from these text files, combined with parameters for data quality assessment by MassyTools software version 1.0.2 for python 3 build 200129a.^46^ The extraction window ranged from 0.00596 to 0.11493 according to the formula ‘(0.00003 ∗ "m/z") - 0.02690’.^34^ Spectra were excluded when any of four parameters was below the mean minus 2 times the standard deviation: the total intensity of a spectrum, the fraction of analyte area with signal-to-noise (S/N) above 9, the fraction of analyte area minus background area above signal-to-noise 9, and the fraction of the spectrum contributed by the analytes. Analyte curation was performed based on quality criteria minima: S/N had to be greater than 9, the deviation of the observed from the theoretical isotopic pattern (isotopic pattern quality or IPQ) less than 0.2 (i.e. <20% deviation) and absolute PPM-error lower than 10. Each of these parameters was evaluated per biological group. A glycan was included if at least 25% of all spectra per biological group passed these quality criteria. As a final curation step, each spectrum had to contain at least 50% of the passed analytes, otherwise the spectrum was removed. A few technical duplicate measurements were performed, of which the one with higher total intensity was selected for analysis. Glycan relative abundances were calculated by normalizing the area of the respective glycan to the total area of all curated glycans per spectrum. Glycosylation traits were calculated as published previously,^34^^,^^40^^,^^51^ with the calculations to obtain these shown in Table S3.

### Quantification and statistical analysis

All calculations and statistical analyses were performed in the R programming language, version 4.3.2 (R Foundation for Statistical Computing, Vienna, Austria) and Rstudio software, version 2023.12.1 (Posit Software, PBC, Boston, MA).^44^ To monitor the quality of the sample preparation and mass spectrometry measurements, repeatability was tested using a commercially available normal control plasma pool (VisuCon-F), monitoring the ten most abundant glycans (Figure S5). Repeatability of the analyses was good, with CVs ≤1.6% for the ten most abundant glycosylation traits.

A Principle Component Analysis (PCA) was performed to explore the variation in the data (Figure S1). To this purpose, the data were log_10_ transformed and Unit-Variance (UV)-scaled before PCA analysis was applied. Subsequently, cases were compared to controls both at baseline (T0) and at highest severity (TH) using a Wilcoxon rank sum test. The severity score is based on the 4C mortality score,^52^ as described previously,^18^ resulting in a severity scale between 0 and 17. To distinguish the post-hospitalization, control samples and the plasma pool from samples obtained during the hospitalization period, these were assigned a negative severity score. Several representative analytes were presented in boxplots (Figures 2 and 3; Figures S3 and S4). To select the largest significant changes, the results of the Wilcoxon rank sum tests (Table S4) were plotted against the log_2_(fold-change) in a Volcano plot with the absolute fold change cut-off set to 1.5 (highest/lowest group ratio; Figure 2A; Figure S3A). Glycans and glycosylation traits that were matching the above criteria (*p*-value <0.05; fold-change ≥ 1.5) were then used to select the most predictive analytes in case-control grouping, using the automated feature selection by the SES algorithm of the MXM R-package^45^ and displayed in a Receiver Operating Characteristics (ROC) curve (Figures S2B and S3B). This algorithm takes collinearity into account and therefore the selected features may deviate from the ones with lowest *p*-value and highest fold-change.

The data were also analyzed in relation to disease severity. To this purpose, patients were categorized into three severity groups: 0-5 (mild), 6-11 (intermediate), 12-17 (severe). A comparative analysis was performed using a Kruskal-Wallis test, which in case of significance, was followed by the post-hoc Dunn’s test (Figure 3; Figure S3). Correlation between either the severity score or Total IgG bisection and the analyte or glycosylation trait relative abundance was calculated using Spearman’s correlation with R_S_- and *p*-values reported in the respective plots (Figures 3B and 4). Correlation with liver injury markers was also calculated using Spearman’s correlation (Table S6).

The results of all statistical tests were corrected for multiple testing using the Benjamini-Hochberg procedure for each individual statistical question after which a *p*-value <0.05 was regarded as significant.

## Supplemental information

Document S1. Figures S1–S5

Table S1. Detailed participant characteristics, sample information and curated data, related to STAR Methods Lines shaded in red were included in the PCA analysis. However, this same analysis identified these three samples as possible outliers (all from case identified as P084) and were excluded from further analysis. Participant number starting with P=Patient, C=Control, V=VisuCon. Participant numbers match with our previous study on IgG in relation to COVID-19 [S1]. Datapoints can be matched by 'Timepoint'. None of the participants had received a COVID-19 vaccine, however treatment regime as well as diagnostic methods varied between the two waves. For instance, hydrochloroquine was frequently used in the first wave, while patients received corticosteroids such as dexamethasone during the second wave. Criteria for hospital admission also differed between the waves, mainly determined by capacity and national guidelines, and PCR-testing was more frequently used during the second wave, possibly resulting in earlier diagnosis.

Table S2. Analyte list (including two dummy glycans) and relevant properties, related to STAR Methods Analyte compositions with (theoretical) *m/**z*-values and extraction windows. All species are calculated as [M+Na]+. Calibrants used for internal calibration by MassyTools are colored red. The most abundant isotope of the isotopic envelope was used for calibration, in the case that the second isotope is the most abundant it is indicated by (i2). H = hexose; N = N-acetylhexosamine; F = deoxyhexose (fucose); L = lactonized N-acetylneuraminic acid (α2,3-linked); E = ethyl esterified N-acetylneuraminic acid (α2,6-linked). Calculation of the extraction window per analyte for MALDI-FTICR-MS data according to the formula 0.00003 ∗ "m/z" - 0.0269 [S2]. This list was used to extract peak areas, associated with parameters for glycan relative quantification and data quality assessment by MassyTools from the recorded spectra. All glycans not included in the final analysis are shaded gray.

Table S3. Glycosylation traits calculation formulas, related to STAR Methods H = hexose; N = N-acetylhexosamine; F = deoxyhexose (fucose); Fa = Antenna fucose; S = N-acetylneuraminic acid (Sialic acid); L = lactonized N-acetylneuraminic acid (α2,3-linked); E = ethyl esterified N-acetylneuraminic acid (α2,6-linked); M = Mannose; Hy = Hybrid; A = Antenna; T = Total; C = Complex.

Table S4. Case-control comparison, related to Figures 2 and S2 Results of the Wilcoxon rank-sum test for Timepoint 0 for controls (n = 12) and cases (n = 152). ^1^Indicated significance is based on B-H corrected *p*-value.

Table S5. Receiver Operating Characteristics analyses, related to STAR Methods and Figures S2 and S3 Receiver Operating Characteristics (ROC) analysis was performed for the Case-Control comparison at timepoint of hospital admission (T0) and at highest severity (TH). Feature selection was achieved using the SES algorithm of the MXM R-package [S3] applied to Log10-normalized and scaled data on the significant glycans and traits from the Wilcoxon tests with sufficient fold change (1.5). This algorithm could in theory output multiple sets of covariates for a single comparison. This happened for both T0 and TH, therefore two models for both timepoints are included in the table. For each of these sets of covariates a general logistic model was calculated and used in a 10-fold cross validation procedure. From this cross-validation procedure, the Area Under the Curve (AUC) was calculated. As a comparison the single parameter models are also included.

Table S6. Correlation analysis, related to STAR Methods Spearman’s correlations between alanine aminotransferase (ALAT), aspartate aminotransferase (ASAT) and the glycans and glycosylation traits, with corresponding *p*-values at time of hospitalization (n = 99) and at time of highest disease severity (n = 128). Adjusted *p*-values are according to Benjamini-Hochberg multiple testing correction per liver injury marker per timepoint. Significant correlations are highlighted in green.
