# Supplementary material for: Total plasma N-glycomic signature of SARS-CoV-2 infection
Source: iScience. 2024 Jun 24;27(7):110374. doi: 10.1016/j.isci.2024.110374 (PMC11294702; doi:10.1016/j.isci.2024.110374)
Supplement: Document S1. Figures S1–S5 [file mmc1.pdf]

iScience, Volume *27*

Supplemental information

Total plasma *N*-glycomic signature of SARS-CoV-2 infection

Marco R. Bladergroen, Tamas Pongracz, Wenjun Wang, Simone Nicolardi, Sesmu M. Arbous, Anna Roukens, Manfred Wuhrer, BEAT-COVID group, and LUMC COVID-19 group

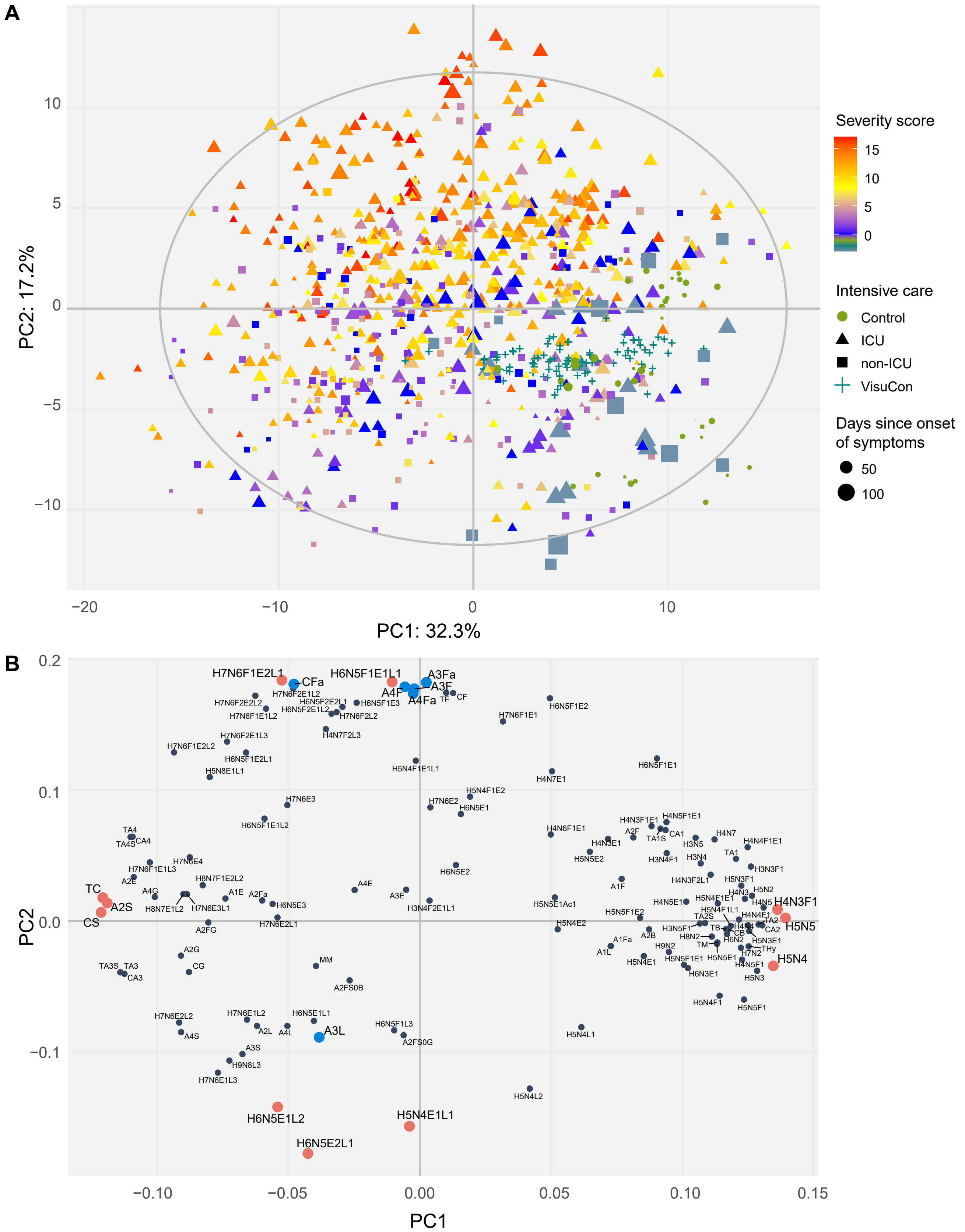

Figure S1: Principle Component Analysis, related to STAR Methods.
A) Scoreplot illustrating the gradient in severity score embedded in the data. Controls as well as 6-week post hospital release are clearly distinguishable from high severity cases. Days since onset is illustrated by the size of the datapoints, severity score is indicated by the color gradient. Missing severity scores (Controls, VisuCon and 6-week post hospital release) were assigned negative values to distinguish them from measured values. Each dot represents a sample (multiple dots per individual). PC1 and PC2 refer to principle components 1 and 2. Percentages indicate contribution to data variability. **B**) Loadingsplot. In orange, three outermost traits in either PC direction are shown, assuming these as having a large influence on the model. In light blue, traits with most influence on the model as calculated using the modelling power.

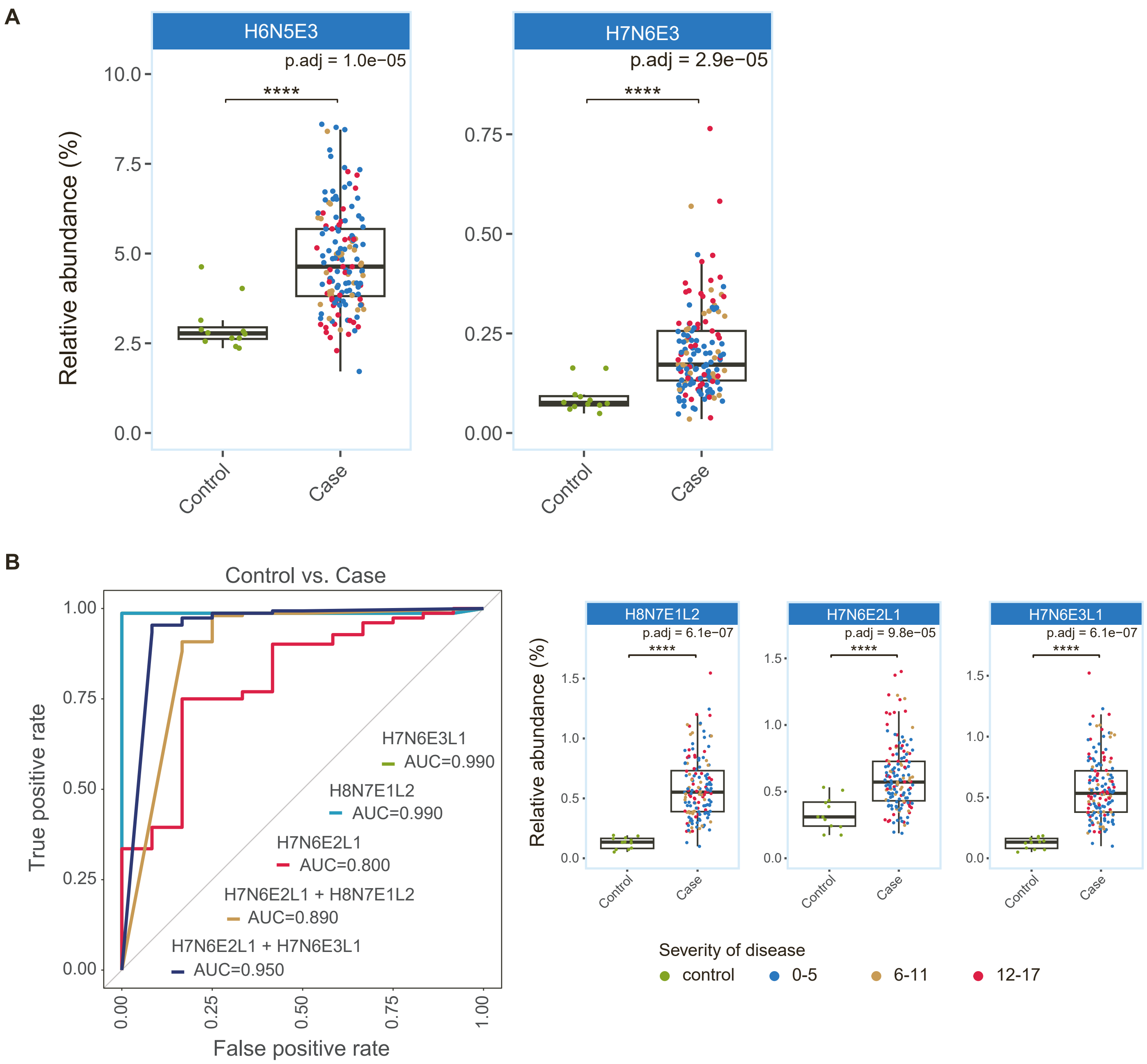

Figure S2: Differentially expressed glycans in COVID-19 patients at time of hospital admission (T0), related to Figure 2.

A) In addition to Figure 2, two more 2,6-sialylated, non-fucosylated glycans showing a distinct relative abundance between COVID-19 patients and healthy controls are shown, indicating major differences in linkage-specific sialylation of TPNG. **B**) Severity prediction in COVID-19 patients: ROC-curves showing two related models distinguishing COVID-19 patients from healthy controls. For clarity and ease of use, the concurring boxplots from Figure 2 are repeated in this figure. The three glycans were selected using the SES-algorithm of the MXM R-package, shown with the dark blue and brown lines. The Spearman correlation test for H7N6E2L1 vs. H8N7E1L2 (S = 186192, p-value < 2.2e-16, rho=0.7467223) as well as H7N6E2L1 vs. H7N6E3L1 (S = 184152, p-value < 2.2e-16, rho=0.7494974) indicate a high correlation between the three glycans. We therefore hypothesize that patients can be distinguished from healthy controls by H8N7E1L2 or H7N6E3L1 alone. The ROC curve for H7N6E3L1 (green line) is not visible, since it overlays exactly with that of H8N7E1L2. Of note, the models might be somewhat inflated because of class imbalance.

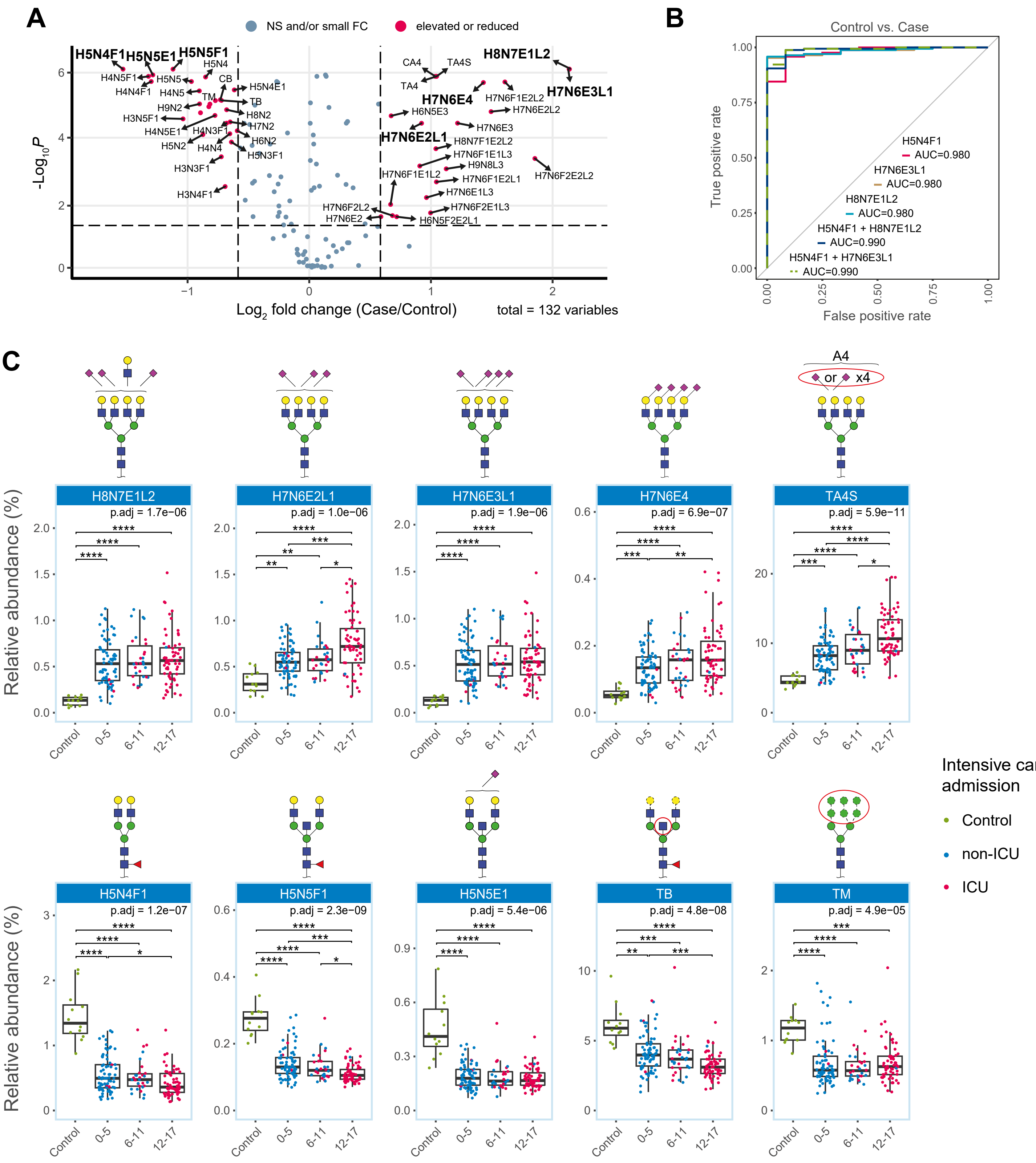

Figure S3: Differentially expressed glycans and glycosylation traits in COVID-19 patients at highest severity (TH), related to Figure 2.

A) Volcanoplot showing the significantly reduced and increased glycans and glycosylation traits in COVID-19 patients compared to healthy individuals. Values on the x- and y-axes show log_2(fold-change) vs. $-log_{10}$(p-value) of a Wilcoxon rank-sum test respectively after Benjamini-Hochberg multiple testing correction (α=0.05). Fold-change cut-off is set to 1.5. **B**) ROC-curves showing two related models distinguishing COVID-19 patients from healthy controls. Both combined models performed equally well. The three glycans in each model were selected using the SES-algorithm of the MXM R-package and illutrated in bold in Figure A. The ROC curves for each individual glycan are also shown. **C**) Boxplots of the most informative glycans and glycosylation traits as deducted from figures A and B, colored according to intensive care admission (at any point in time during hospital admission). Concurrent glycan schemes are illustrative, since the exact isomers are not known. Dashed lines represent optional building blocks.

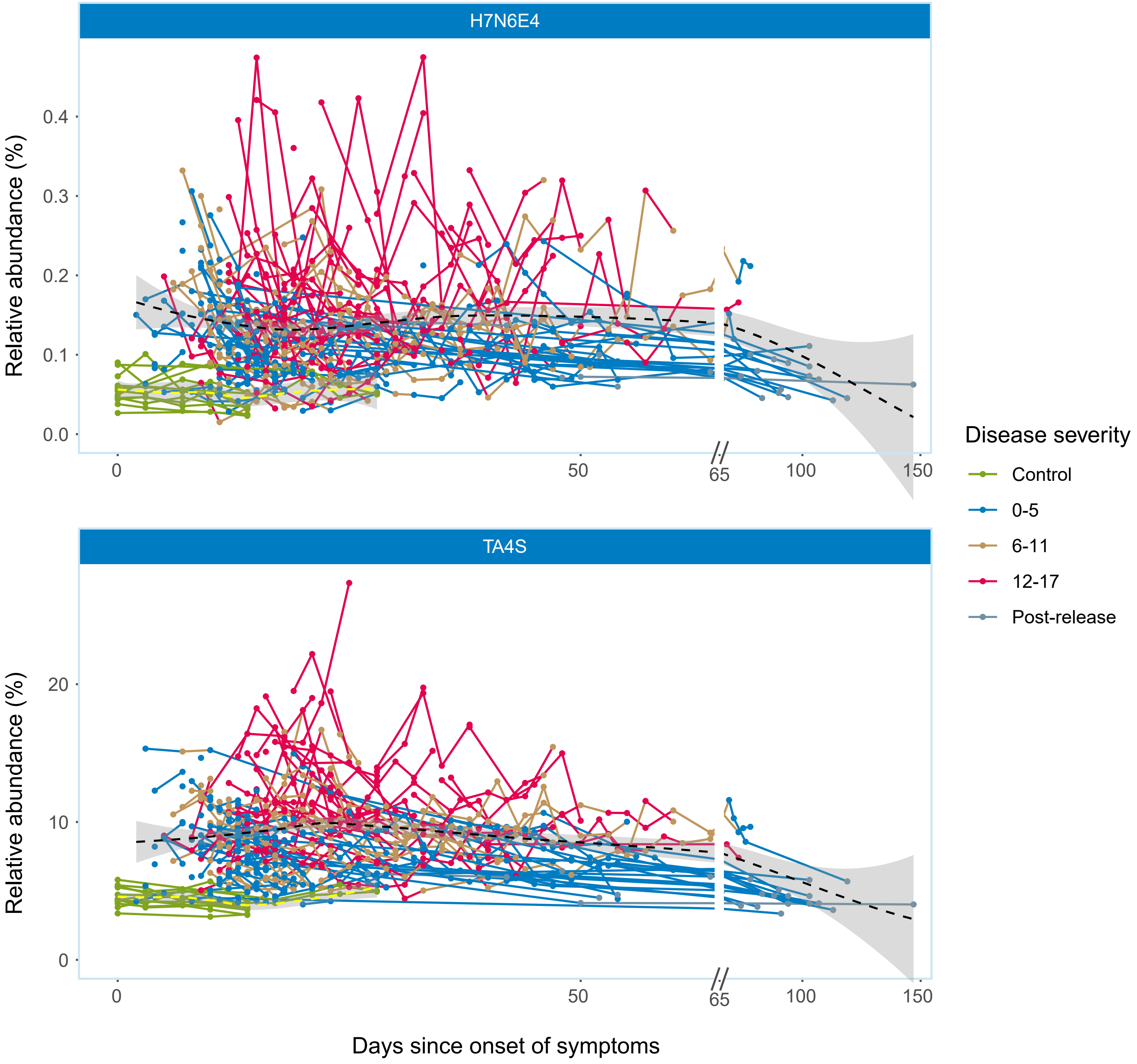

Figure S4: Longitudinal expression of the two most informative traits, related to Figure 3.
Both trendlines for cases (black dashed lines) show a higher level compared to controls (yellow dashed lines) in the first days after onset of disease. TA4S is increasing with development of the disease, with a maximum between 20 and 25 days. Upon treatment and cure, relative abundances seem to revert to default (control) levels. Grey area's represent 95% confidence intervals for the trendlines.

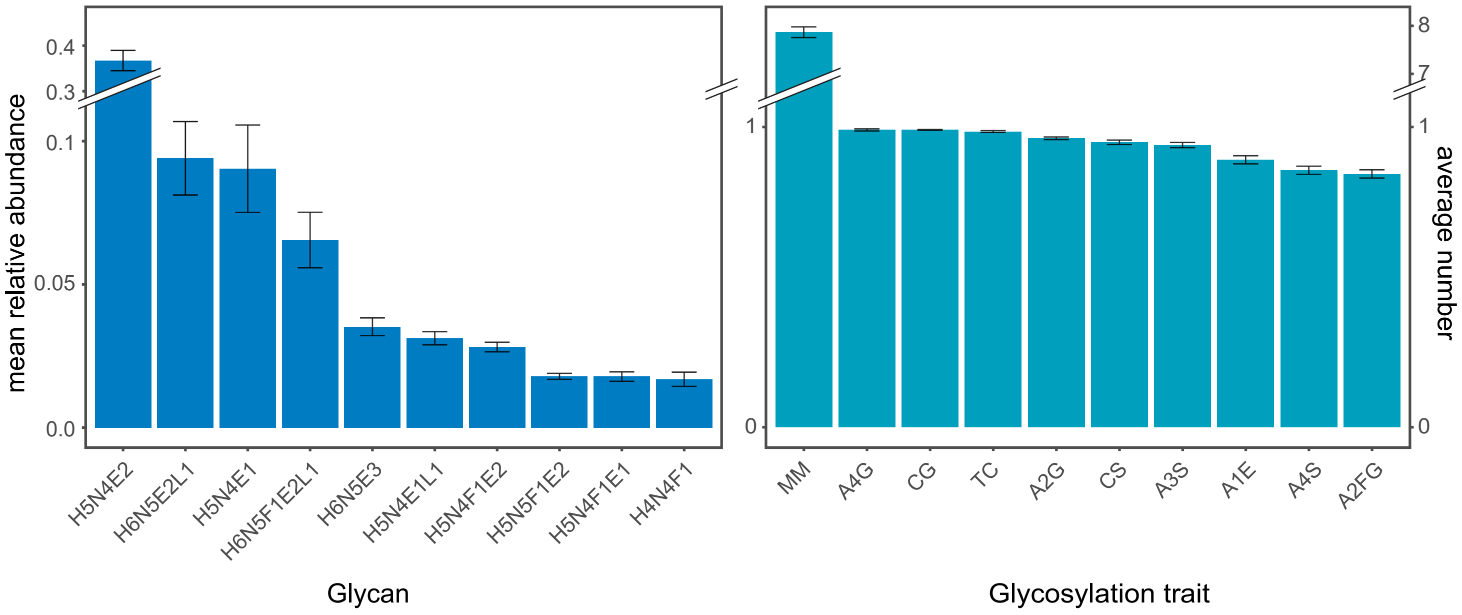

Figure S5: Repeatability using standard plasma (VisuCon), related to STAR Methods.
Data from 89 VisuCon samples. MM means average number of mannose units for the oligomannose type glycans. This is an absolute value (right axis). All other glycans and glycosylation traits are shown as relative abundance (left axis). H = hexose; N = N-acetylhexosamine; F = deoxyhexose (fucose); L = lactonized N-acetylneuraminic acid (α2,3-linked); E = ethyl esterified N-acetylneuraminic acid (α2,6-linked); S = Sialylation; G = Galactosylation; T = Total; A = Antenna; C = Complex.

Supplemental References

[S1] Pongracz, T., Nouta, J., Wang, W., van Meijgaarden, K.E., Linty, F., Vidarsson, G., Joosten, S.A., Ottenhoff, T.H.M., Hokke, C.H., de Vries, J.J.C., et al. (2022). Immunoglobulin G1 Fc glycosylation as an early hallmark of severe COVID-19. EBioMedicine *78*. 10.1016/J.EBIOM.2022.103957.

[S2] Vreeker, G.C.M., Nicolardi, S., Bladergroen, M.R., Van Der Plas, C.J., Mesker, W.E., Tollenaar, R.A.E.M., Van Der Burgt, Y.E.M., and Wuhrer, M. (2018). Automated Plasma Glycomics with Linkage-Specific Sialic Acid Esterification and Ultrahigh Resolution MS. Anal Chem *90*, 11955–11961. 10.1021/acs.analchem.8b02391.

[S3] Lagani, V., Athineou, G., Farcomeni, A., Tsagris, M., and Tsamardinos, I. (2017). Feature Selection with the R Package MXM : Discovering Statistically Equivalent Feature Subsets. J Stat Softw *80*. 10.18637/jss.v080.i07.
